# Supplementary material for: Water-dispersible unadulterated α-mangostin particles for biomedical applications
Source: R Soc Open Sci. 2020 Nov 18;7(11):200543. doi: 10.1098/rsos.200543 (PMC7735336; doi:10.1098/rsos.200543)
Supplement: Supplementary Information for Water-dispersible unadulterated α-mangostin particles for biomedical applications [file rsos200543supp1.pdf]

Supplementary Information for

Water-dispersible unadulterated α-mangostin particles for biomedical applications

Jutamad Bumrung,[a,b] Chanpen Chanchao,[c] Varol Intasanta,[d] Tanapat Palaga[e] and Supason Wanichwecharungruang *[a,b]

[a] Department of Chemistry, Faculty of Science, Chulalongkorn University, Bangkok, Thailand

[b] Center of Excellence in Advanced Materials and Biointerfaces, Chulalongkorn University, Bangkok, Thailand

[c] Department of Biology, Faculty of Science, Chulalongkorn University, Bangkok, Thailand

[d] National Nanotechnology Center, National Science and Technology Development Agency, Pathumthani, Thailand

[e] Department of Microbiology, Faculty of Science, Chulalongkorn University, Bangkok, Thailand

Table S1. Various conditions used in the preparations of α-mangostin particles

Table S2. The clear inhibition zone against *P. acne* of original α-mangostin and MG-E.

Fig S1. Size distribution in water of (a) MG-E (b) MG-P and (d) MG-B.

Fig S2. NMR spectrum of original α-mangostin, MG-E, MG-P and MG-B.

Fig S3. The clear inhibition zone against *P.acne* of original α-mangostin and MG-E.

Table S1. Various conditions used in the preparations of α-mangostin particles

#	α-Mangostin (mg)	Ethanol (mL)	Starting [α-mangostin] in ethanol (%w/v)	Water (mL)	Final [α-mangostin] in the obtained suspension (%w/v)	Amount of ethanol (%v/v) in water
1	25.0	1.0	2.5	4.0	0.5	20.0
2	50.0	1.0	5.0	4.0	1.0	20.0
3	250.0	1.0	25.0	4.0	5.0	20.0
4	500.0	1.0	50.0	4.0	10.0	20.0
5	1500.0	1.0	150.0	4.0	30.0	20.0
6	25.0	0.025	100.0	4.80	0.5	0.5
7	25.0	0.25	10.0	4.75	0.5	5.0
8	25.0	1.0	2.5	4.0	0.5	20.0
9	25.0	2.5	1.0	2.5	0.5	50.0

Table S2. The deconvoluted peak area of original α-mangostin, MG-E and MG-B.

	The deconvoluted peak area (%)			
	C=C	C-C	C-O-C and C-OH	C=O
original α-mangostin	57.09	35.58	4.70	2.63
MG-E	53.62	33.84	10.50	2.04
MG-P	55.95	33.38	8.66	2.00
MG-B	55.38	33.49	9.14	1.98

Table S3. The clear inhibition zone against *P. acne* of original α-mangostin and MG-E.

Concentration (mg/mL)	Clear inhibition zone (mm)	
	original α-mangostin	MG-E
1.25	11.83 ± 0.29	12.33 ± 0.58
2.50	12.83 ± 0.29	13.50 ± 0.50
5.00	14.50 ± 0.50	15.33 ± 0.58
10.00	15.17 ± 0.76	16.50 ± 0.50
20.00	16.83 ± 0.29	17.17 ± 0.29

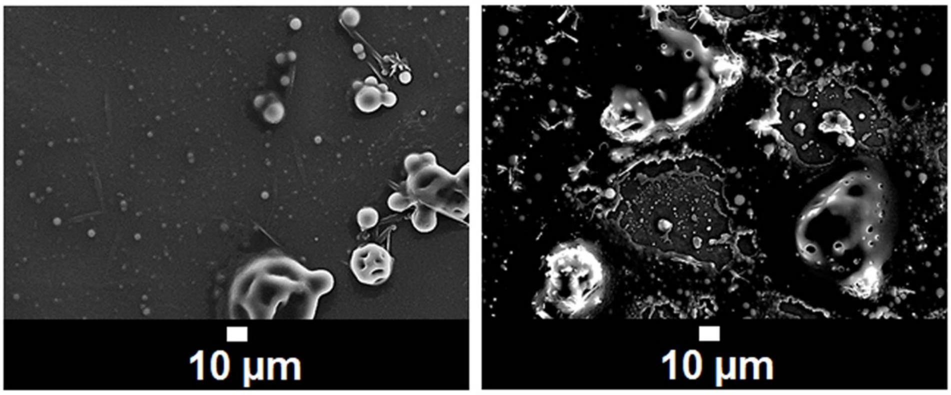

Figure S1. SEM images of MG particles prepared at starting α-mangostin concentrations of 10% (left) and 30% (right) w/v.

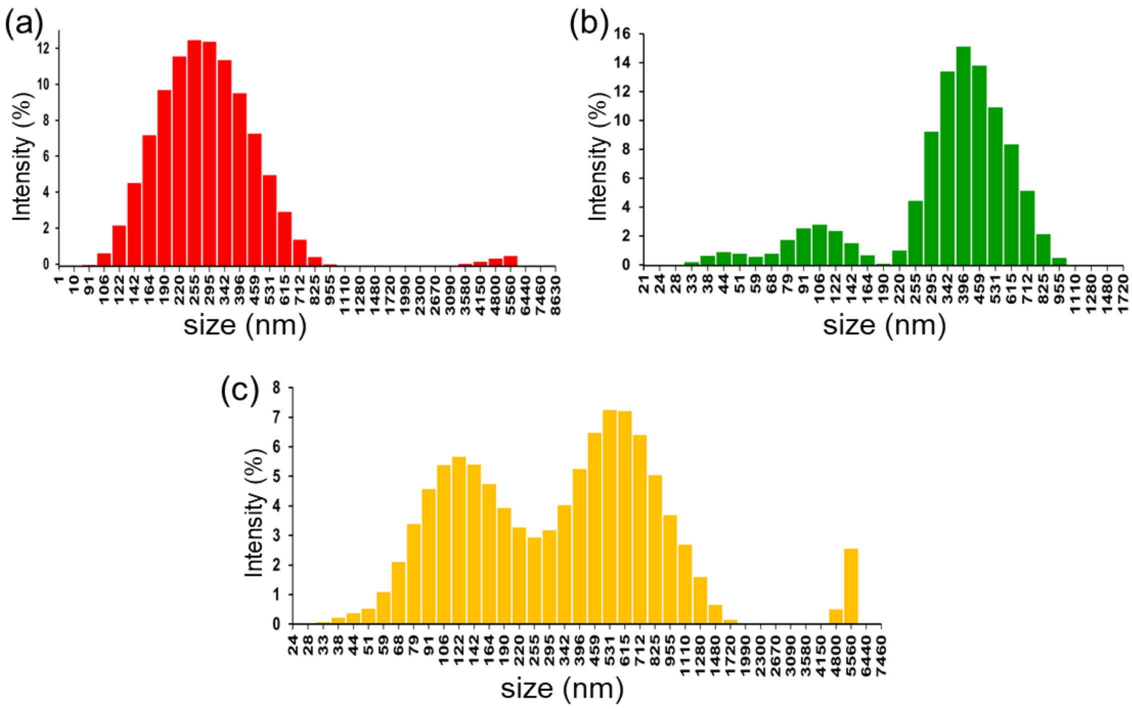

Fig S2. Size distribution in water of (a) MG-E (b) MG-P and (d) MG-B.

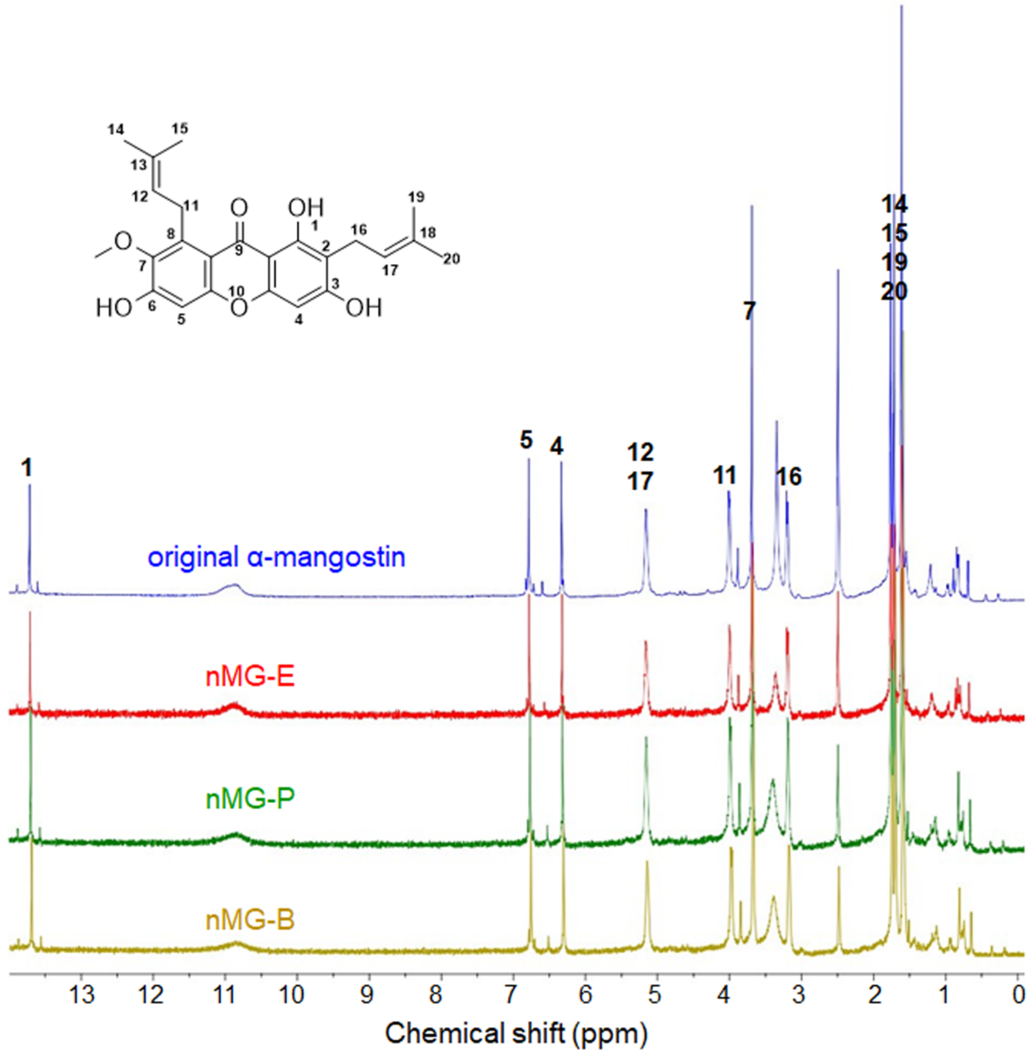

Fig S3. NMR spectrum of original α-mangostin, MG-E, MG-P and MG-B.

α-Mangostin particles obtained from process using ethanol (MG-E): ^{1}H NMR (400 MHz, DMSO-d_6, δ ppm) 1.59 (s, 6H, 19- and 20-CH_3), 1.70 (s, 3H, 15-CH_3), 1.75 (s, 3H, 14-CH_3), 3.18 (d, *J* = 7.0 Hz, 2H, 16-H), 3.67 (s, 3H, 7-OMe), 3.99 (d, *J* = 6.2 Hz, 2H, 11-H), 5.15 (s, 2H, 12- and 17-H), 6.31 (s, 1H, 4-H), 6.77 (s, 1H, 5-H) and 13.70 (s, 1H, C-1-OH). ATR FT-IR (cm^{-1}): 3385.9 (brd, O-H stretching), 2962.5, 2914.5, 2858.3 (m, C-H stretching of saturated carbons), 1643.4 (str, C=O stretching of aromatic ketone), 1605.1, 1580.4 (str, C=C stretching of aromatic ring), 1461.4, 1432.6 (str, C-H bending of methyl groups), 1373.4 (str, C-H bending of gem dimethyl), 1278.2 (str, C-O-C stretching of methoxy group on benzene ring), 1226.8 (str, C-O stretching of vinyl ether), 1184.1, 1154.2 and 1078.7, 1044.1 (str, C-O stretching of C-O connected on hydroxyl groups). UV-Visible absorption (λ_{max} nm in ethanol): 248 (π - π* transition) and 321 (n - π* transition).

α-Mangostin particles obtained from process using propanol (MG-P): ^{1}H NMR (400 MHz, DMSO-d_6, δ ppm) 1.59 (s, 6H, 19- and 20-CH_3), 1.70 (s, 3H, 15-CH_3), 1.74 (s, 3H, 14-CH_3), 3.17 (d, *J* = 6.4 Hz, 2H, 16-H), 3.67 (s, 3H, 7-OMe), 3.98 (d, *J* = 6.2 Hz, 2H, 11-H), 5.14 (s, 2H, 12- and 17-H), 6.30 (s, 1H, 4-H), 6.76 (s, 1H, 5-H)

and 13.69 (s, 1H, C-1-OH). ATR FT-IR (cm^{-1}): 3393.5 (brd, O-H stretching), 2963.1, 2913.0, 2856.9 (m, C-H stretching of saturated carbons), 1642.6 (str, C=O stretching of aromatic ketone), 1605.3, 1579.9 (str, C=C stretching of aromatic ring), 1460.7, 1432.5 (str, C-H bending of methyl groups), 1373.5 (str, C-H bending of gem dimethyl), 1278.1 (str, C-O-C stretching of methyl groups on benzene ring), 1225.6 (str, C-O stretching of vinyl ether), 1184.0, 1154.3 and 1077.9, 1044.0 (str, C-O stretching of C-O connected on hydroxyl groups). UV-Visible absorption (λ_{max} nm in ethanol): 248 (π - π* transition) and 321 (n - π* transition).

α-Mangostin particles obtained from process using butanol (MG-B): ^{1}H NMR (400 MHz, DMSO-d_6, δ ppm) 1.59 (s, 6H, 19- and 20-CH_3), 1.70 (s, 3H, 15-CH_3), 1.74 (s, 3H, 14-CH_3), 3.17 (d, *J* = 6.7 Hz, 2H, 16-H), 3.67 (s, 3H, 7-OMe), 3.97 (d, *J* = 6.7 Hz, 2H, 11-H), 5.14 (s, 2H, 12- and 17-H), 6.30 (s, 1H, 4-H), 6.76 (s, 1H, 5-H) and 13.69 (s, 1H, C-1-OH). ATR FT-IR (cm^{-1}): 3394.3 (brd, O-H stretching), 2963.0, 2923.4, 2857.8 (m, C-H stretching of saturated carbons), 1642.2 (str, C=O stretching of aromatic ketone), 1605.3, 1579.8 (str, C=C stretching of aromatic ring), 1460.3, 1432.4 (str, C-H bending of methyl groups), 1373.6 (str, C-H bending of gem dimethyl), 1278.1 (str, C-O-C stretching of methyl groups on benzene ring), 1225.9 (str, C-O stretching of vinyl ether), 1184.1, 1154.2 and 1078.1, 1043.9 (str, C-O stretching of C-O connected on hydroxyl groups). UV-Visible absorption (λ_{max} nm in ethanol): 248 (π- π* transition) and 321 (n - π* transition).

α-Mangostin (original raw material): ^{1}H NMR (400 MHz, DMSO-d_6, δ ppm) 1.60 (s, 6H, 19- and 20-CH_3), 1.70 (s, 3H, 15-CH_3), 1.75 (s, 3H, 14-CH_3), 3.18 (d, *J* = 6.8 Hz, 2H, 16-H), 3.68 (s, 3H, 7-OMe), 3.99 (d, *J* = 5.9 Hz, 2H, 11-H), 5.15 (s, 2H, 12- and 17-H), 6.32 (s, 1H, 4-H), 6.77 (s, 1H, 5-H) and 13.70 (s, 1H, C-1-OH). ATR FT-IR (cm^{-1}): 3142.2, 3246.3 (brd, O-H stretching), 2962.0, 2913.9, 2855.7 (m, C-H stretching of saturated carbons), 1640.1 (str, C=O stretching of aromatic ketone), 1607.3, 1581.4 (str, C=C stretching of aromatic ring), 1450.9 (str, C-H bending of methyl groups), 1372.8 (str, C-H bending of gem dimethyl), 1328.4 (wk, O-H bending), 1276.1 (str, C-O-C stretching of methoxy group on benzene ring), 1238.2, 1222.8 (str, C-O stretching of vinyl ether), 1199.1, 1182.4, 1169.2 and 1094.9, 1075.1, 1049.9 (str, C-O stretching of C-O connected on hydroxyl groups). The maximum absorption peak (λ_{max}, ethanol, nm): 248 (π - π* transition) and 321 (n - π* transition).

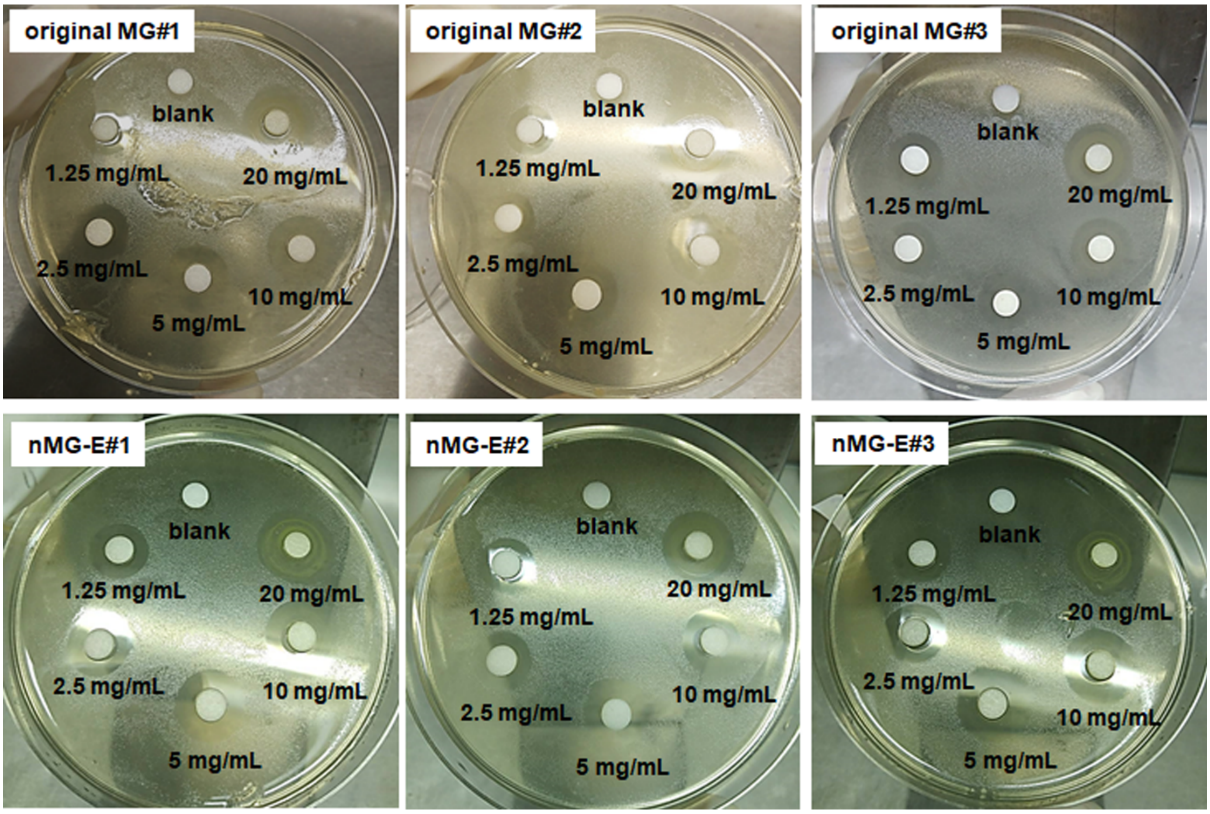

Fig S4. The clear inhibition zone against *P. acne* of the original α-mangostin and MG-E.
